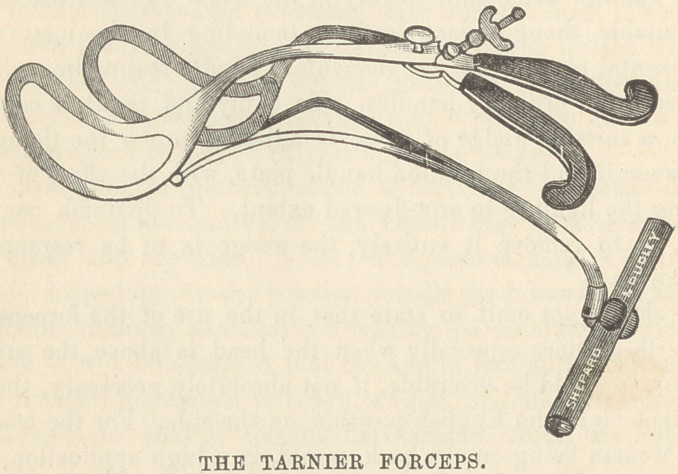# The Mechanics of the Obstetrical Forceps

**Published:** 1880-03

**Authors:** John Bartlett


CHICAGO MEDICAL

JOURNAL & EXAMINER.

VOL. XL.—MARCH, 1880.—NO. 3.

[In these pages dimension and weight are expressed in terms of the Metric System, and temperature in degrees of the Centigrade Scale.]

### Original Communications.

ARTICLE I.

### THE MECHANICS OF THE OBSTETRICAL FORCEPS. A Thesis presented to the Chicago Gynæcological Society, by JOHN BARTLETT, M.D., January 16, 1880.

MR. PRESIDENT: I have announced for my subject this evening The Mechanics of the Obstetrical Forceps. While it is not my intention to go elaborately into the subject, I shall offer some remarks on the several portions of the instrument.

#### THE HANDLES.

The first forceps of which we have a reliable report, made by Chamberlen, had a handle like those of scissors, and the first instrument described and figured in obstetrical literature, that of Giffard, noticed by Chapman in 1739, had one of iron forming a hook, the point of which was turned toward its fellow. A few years later Smellie adopted the handle of iron and wood, which has served as a model to the present day.

The thin iron handles of Giffard are also now in use in a modified form, as may be seen in the standard instruments of Hodge, Wallace and others. And this, notwithstanding that a worse form for a handle can hardly be conceived: in no department of mechanics or art, probably, can there be found a tool intended for the powerful grasp and traction of the hand, made of small, polished, springing rods of steel terminating, as a provision against slipping, in yet smaller rods, set at an angle to the others. In practice they fail to furnish a good hold for the hand in adjusting the instrument, and as a means of traction they are even more faulty. I can conceive of nothing but imitation which has kept such models so long in existence.

### THE SHANKS.

The relation of the handle to the shank in the majority of instruments is that of a continuous, or nearly continuous line. The handles of Hodge's forceps are depressed slightly below the shanks in order to "preserve the line of traction of the blades." The handles of the forceps of Bedford, Aveling and Fitch are curved more or less downward, the latter two furnishing an admirable pistol-handle grip.

The shanks of the earlier forceps formed part of the cephalic curve in diverging from one another near the lock; later, as in Hodge's forceps, the head curve was formed almost entirely by the blades, the shanks being more or less parallel to one another. The object of this change was to secure a more easy application of the clams to the head, seeing that the mother's tissues permitted of a freer movement of instruments so formed and tended less by pressing forward the shanks to push or tilt the blade out of position. The new shape also was intended to render less decided that unnatural form of stretching of the perineum observed when it is compelled to span over the wide space between the shanks of forceps of the older style, as the head nears the vaginal outlet.

### THE BLADES.

The blades of the forceps first made by Chamberlen have served as a model for all others. Various minor changes in the cephalic curve have been made. This curve, I believe, in almost all forceps, has been adapted for that position on the head which it most generally assumes in low deliveries. The more recent instruments, however, as that of Wallace, are apparently constructed, as they should be, not only with reference to fitting the head as presenting low in the pelvis, but also with regard to seizing and holding it when at or above the superior strait. The internal lateral curve of some forceps, by which I mean the curve from side to side on the inner face, as in that of Dr. Davis', imitated in the genuine Hodge's instrument, is so marked that we may assume that the originator counted largely upon it, as a species of counter-sinking, for making firm the grasp of the head. It would be desirable to form this face of the blade to correspond as nearly as possible with that portion of head and face to which it is more generally applied. From measurements made by myself, it would appear that this internal lateral curve near the heel of the blade should correspond to a segment of a circle having a radius of 2½ inches. While near the point, the curve should be one of 4½, and about the middle of the clam 3 inches radius. The external lateral curves embraced by the walls of the parturient canal, should correspond to those of the internal curve, the thickness of the clams at the several points of measurement being, of course, added to the several radii above given.

The pelvic curve, invented, as is generally supposed, by Levret and Smellie, in 1747–1851, is the most important of the changes made in the instrument since its origination by the Chamberlens. It has been variously modified so that while some recently devised forceps have a curve as high as 4½ inches, the majority of practitioners seem, in their selection of instruments, to indorse the statement of Barnes, that the curve should be moderate. The fact is, as will appear further on, that while a high curve seems desirable, as ordinarily used, forceps so constructed have not a good hold, or they have an inconveniently wide blade, and all of them tend to slip off in the direction of a line joining the resistance and the power.

### THE MANNER AND CAUSE OF SLIPPING OF THE BLADES.

The forceps may slip off in the direction of the cephalic curve, by the opening or springing of the points of the blades; or the blades may be drawn off the head while yet in a fair position to hold, in a line more or less departing from their long axis. In the one case the head may be represented as slipping through the yielding ends of the blades; in the other as slipping between their edges. The cause of the one of these modes of slipping may be too slight a cephalic curve, or too weak a blade. The cause of slipping through the blades' edges is traction in a line out of their longitudinal axes—usually the result of drawing upon curved forceps in a line parallel to their shanks, deviating more or less from the axes of the blades, according as the pelvic curve may be higher or lower.

The blades of Smellie's forceps had a moderate width. When the second curve was added, the direction of traction falling at more or less of an angle with the blades, developed a tendency to slip off the head sidewise, as explained by Chapman in his (the first) description of the forceps. Doubtless the new pelvic curve was one of the causes of the insecure hold of Giffard's forceps, which forced Chapman, as he says, to lay the instrument aside, and resort to turning and the fillet for ten years.

In this connection I may cite the statement of an older writer, to the effect that Levret's curve, as first devised, was more considerable than that of his latest instruments. We may safely presume that once the conception of conforming the forceps to the curve of the pelvis having entered the mind of Levret, he proceeded to take the pelvic curve as a guide. Then we can as easily imagine that when he came to use the instrument, he quickly discovered that a pronounced curve had the fatal disadvantage of allowing of lateral slipping, and that finally he compromised between the necessity for a curve and the inconveniences of a considerable one, by adopting that one which has retained its place for more than a hundred years.

To counteract the tendency to slipping off sideways, we may suppose that Dr. Robert Wallace Johnson, who wrote in 1768, devised his peculiar forceps, the lower rim of the blade of which projected awkwardly backward, forming the so-called perineal curve, so that one rim of the instrument might rest in front of the parietal protuberance, while the other took strong hold behind it. This instrument was followed by the Davis forceps, constructed with like intention, and this latter blade, variously modified, has come down to the present generation in the standard instruments of Hodge, Wallace, Smith and others.

### THE LOCK.

The lock of the forceps has frequently been changed since the time of Chamberlen, and not always for the better. He tied his blades together with a thong, passing through holes suitably located. Giffard, or some one before him, thought to improve upon this by substituting a screw. This was discarded by Chapman; and as I regard the setting aside of this mode of connecting the branches, and the final substitution of a different method, as of particular interest, I beg leave to introduce some extracts from the Midwifery of Dr. Chapman, published in 1739.

Please to observe with what emphasis he declares that his instrument held very much better, and was applied with greater ease to the patient and himself, when the blades were loosely interlocked and held together with the hand, than when stiffly joined by the screw.

"When the blades are passed they are then to be brought together, and, if you please, the screw may be put through and fastened with the button, though there is no occasion for the loss of so much time, for without doing this the hand will prove sufficient to keep them together." (It is necessary here to explain that in Giffard's forceps, which Chapman used, there was a recess in one blade at the lock into which the other rested as we see in some pincers used by artizans.)

"It is much better" continues Chapman, "that the forceps should not be joined or fixed by a screw, because when they are not screwed together, though they should not happen to be exactly opposite to each other, yet they will turn so as to take fast hold of the head and readily extract it."

"I have always found the instrument far less apt to slip since I omitted fastening (screwing) the parts together; and with more ease to the patient as well as myself, and in much less time than before, have found the head of the child fairly fixed in the instrument."

"Mr. Giffard frequently complains that his extractor (forceps)

slipped, which I am fully persuaded it would not have done if the parts had been left unjoined (unscrewed) as I now use them."

Subsequently to this writing Dr. Chapman (according to Rigby) invented that method of uniting the parts of forceps which has come down to us as the English lock, though it is to be regretted that the efficiency which its originator strove to give it has been and is now too often marred by changing it from a loose to a tight lock. The lock of Siebold known as the German, as well as the pivot lock, known as the French, and all the modifications of it which have fallen under my notice are greater or less improvements upon the Giffard lock.

I shall speak presently of the part which the lock plays in securing thorough opposition of the blades and a firm hold; and before reaching that subject I wish to call attention to a lock recommended by Dr. Bond in the *American Journal of Medical Sciences* for 1850, and described and figured in *Churchill's Midwifery.* The very stout and peculiarly shaped screw of this lock was designed by its inventor to allow the rocking of the blades from side to side about a line passing centrally from heel to point. This lock is perfectly adapted to such a purpose, and it would, without doubt, with some modification, admit of a see-saw motion of the blades on the lock as a pivot and in parallelism with the central plane of the instrument, and what, as will be seen, is of great importance, this lock would regulate both the rocking and the other motion, which I shall call racking, so that both movements may be limited within proper bounds.

Some writers express a preference for the English lock because it is simple and easy to lock and unlock. A few, as J. Y. Simpson, take note of the essential point, that it should be loose. It is not such a lock as this (showing forceps with a very close lock) that Chapman recommended, but such as this (showing a genuine Simpson's forceps).

Before following me into the study of the advantages of this lock, please note particularly its characteristics. Observe the ends of the handles while I rack them by one another, when closed. You see that they have a play so considerable that the upper surface of the right handle nearly corresponds to the lower surface of the left, and the reverse. Now observe the points of the blades while the handles are racked as before and note how one plays by the other in the plane of the handles. Now remark the motion of a blade when a movement of rotation is imparted to its handle.

I desire to call your attention to the advantages of these movements and I will illustrate what I have to say on these forceps, known as the instrument of Dr. Zeigler or Dr. Barclay; for in them there is such an exaggeration of the movements to which I wish to call attention, that they cannot fail to be noted all over the room.

I will first state the advantages of the loose lock and then illustrate them with the instruments and model,

1. The loose-lock forceps are easier of application. 2. They are less apt to injure the head of the child. 3. They can be used with less pain to the patient. 4. They are less liable to injure the tissues of the mother. 5. A less amount of traction is necessary with them than with other varieties. 6. They hold the head seized in whatever diameter or direction, with a powerful grasp. 7. In the event of the necessity arising for turning the head in the pelvis, as in rotating the occiput forward, the loose-lock forceps renders the operation safer and easier.

Let us consider the advantages here claimed, seriatim.

(1.) They are easier of application: The greater facility of application has no reference to the introduction or near apposition of the blades, but to their *locking*. What is the obstacle to the locking of the forceps when the blades are very nearly but not quite in apposition? Let us see. Take this stiff-lock instrument, a modern Bedford, and apply it to this head, we will say in the left occipito-cotyloid position. Here rests the right blade, over the occiput and the left over the brow, but not over it exactly, for it rests on, and impinges against, the head by the posterior rim of the blade only. Note the position of the anterior rim, how it projects in front of the head; in this instance quite an inch. Now, we are ready to appreciate one of the causes of difficulty in locking. When not locked but in near apposition, this left blade would rest thus, upon the front and side of the forehead and bound down in that position by the vagina acting like this wide rubber band, which is drawn over the blades. Let us now attempt to lock the forceps. You observe that it cannot be done without stretching the rubber band; that the rotation outward of the blade in an attempt to cause it to assume the required position of exact apposition to its fellow, is resisted by a force proportionate to the strength of this band, or, we may say, of the walls of the parturient canal. In practice, encountering such a resistance when the forceps are as here represented, what do we do? We are told by the experienced to press the handles far backward, and to push the blades upward; why? For this reason; by so doing, we cause the wide part of the blade to move off of, and above the point where the vagina clamped the clam into a wrong position, and in lieu of the widest surface, we substitute as the portion resting on the head, the narrowest part of the blade; then we make an effort at locking. The stretching of the vaginal wall, the degree of lifting of it from the head, necessary for the twisting into position of the blade, is much lessened, and the effort at locking is probably a success. Now, with the loose-lock forceps, *all this difficulty is avoided.* The blades, when introduced, assume exactly the position of those of the stiff-lock instrument just pointed out; but when we seek to lock them there is no difficulty; the loose-fitting lock allows the pronated blade to accommodate itself to the surface of the head, and locking is at once effected, without resistance from the vagina.

(2) It is much less apt to injure the child. Let me recur again to the position of this rigid-lock instrument on the head, at least with reference to one of its blades. See how the entire hold of the clam rests on one narrow rim, and please to observe that just in proportion as the hold is slender must the compression, to render it secure, be increased. Contrast the position and hold of this blade with that of the Barclay forceps. Note how the blades of the latter are equably and closely pressed against the surface of the head, and no comments will be necessary to assure all of the greater safety to the child in the use of this instrument.

(3) It can be used with less pain to the patient. I presume there are few practitioners here who have not remarked, and perhaps been perplexed at, the outcry of the patient, "You are cutting me," at the moment when, after a propitious locking of the instrument high up, we have begun to draw the blades down firmly on to the head, and before we have made any actual extractive efforts. I have been annoyed and puzzled by this statement of the patient, fearing lest I had included some of the mother's tissues in the grasp of the instrument, when the favorable result would show that such could not have been the case. Now, my opinion is that the pain of which the mother so complains is referable chiefly to the distention which the edge of the forceps makes on the vagina in thrusting it away from the head, in the manner which I have just illustrated. As this stretching of the vaginal tissues is avoided in the Barclay forceps, it may be said that it can be used with less pain to the mother.

(4) And for identical reasons it will be admitted that the loose-lock instrument is proportionately less apt to injure the maternal structures.

(5) It follows, also, that a less amount of traction is necessary with this instrument, inasmuch as the blade is not forced to carry before it, as it were, a wave of distended vaginal wall.

(6) That the hold of an instrument grasping the head as it does, with both rims of each blade flat down upon it, accommodating itself to any inequality in the shape of the head, is unusually firm, needs only to be stated to be acquiesced in, but no consideration of the subject would give one who had not tested these instruments an adequate idea of their really remarkable tenacity of grasp. I invite any gentleman interested to seize this model head with any instrument he may choose, let it be never so stout and unyielding in its blades, and to say whether any such instrument has a firmer grasp than the comparatively delicate blades of this straight Barclay forceps. Turn the head in any and every possible and impossible position and you find this instrument ready to grasp it with "an iron jaw."

(7) In the event of a necessity for turning the head, it would prove certainly a safer instrument than the ordinary forceps, because there would be no outjutting blade to drag upon the vagina, and render the rotation more dangerous, difficult and painful. The blades of the forceps have been well likened to a pair of hands. The mechanism of the loose-lock instrument in seizing the head in a diagonal position may be illustrated by extending this comparison, so that the forearms represent the shanks.

Now if we place the hands upon a head with the occiput obliquely forward and toward the right hand, in positions corresponding to the blades of the forceps under consideration, we shall find that the right forearm is on a higher plane than the left, so that the ulnar margin of the right hand is about on a level with the radial margin of the left (representing the displacements of the blades in regard to the horizontal plane of the closed handles) and that the right hand is inclined slightly toward supination, while the left tends toward pronation, representing the "rotation" of the blades.

There is a certain disadvantage attending excessive looseness of the lock. All of its bearings tend to cause the blades to assume the normal position regarding each other, that is, to lessen and to destroy the displacements of rocking and racking.

Now, when the head is seized in a diagonal position, one of the blades is pressing inward and forward against a surface of the head corresponding to a diagonal diameter of its oval. While the other blade is pressing inward and backward at the opposite diameter, thus producing a very strong tendency to turn the head so that the longer diameters of the oval shall coincide with the conjugate diameters of the pelvic canal. Now, where the head lies with the forehead inclined backward, I know of no objection to this forced rotation of the occiput forward. But when, as usually happens with the head high up, the occiput is directed backward, the tendency of these forceps is to rotate it into the hollow of the sacrum, and thus greatly to increase the difficulty of the delivery. This forced rotation of the occiput is a movement so directly contravening the plans of nature, that I should hesitate to apply such an instrument to a head known to present with the occiput inclined backward. This tendency to rotate the head is sometimes manifested in the ordinary forceps, but, for obvious reasons, it is much less marked in them than in those with very loose locks. It would appear, therefore, that the movements in the Ziegler and Barclay forceps may be excessive, while in a genuine Simpson instrument there may be the proper measure of looseness of lock.

The rocking motion of the blades is most effective in the straight forceps, and it becomes less and less efficient as the axis of the blades departs from the axis of the handle. So that in the very high-curved instruments, the movement is but little available, and if in them the rotation be allowed to be considerable, the hold of the instrument is destroyed. For where a rim of the forceps, because of a high curve, lies far to one side of the axis of rotation (the line of the handle) rotation outward, for instance, becomes (the rims being at the extremity of the long arm of the lever), a forcible lifting of the blade from its hold on the head. Fortunately, however, the hold of high-curved forceps with a stiff lock in high deliveries, is good when the curve of the instrument, or resiliency of the perineum is sufficient to allow of a proper grasp of the head. Thus the hold of the ordinary forceps with one blade over the occiput, and the other on the forehead is firm, and a similar hold over an oblique diameter is often strong. For such deliveries, it would seem desirable to retain the firm-lock, whereas, where the head is within reach of an instrument of moderate curve, as Simpson's, or in cases where the head is well down in the pelvis, the advantages of the loose-lock are available. I know no objection to the racking of the blades in instruments of high curve, and I believe the movement is desirable in such forceps. And for these a modification of the lock of Dr. Bond would, I think, be entirely suitable.

THE PRINCIPLE OF DIRECT TRACTION.

So far as I am aware, the first recognition of the departure from the proper line of traction in the use of the forceps with the pelvic curve was made by Levret in the modification of the curve of his instrument from higher to lower. Osiander, in the year 1799, recognized the necessity of applying tractile force in another direction than the line of the shanks. In his instructions for extracting the head, he advised that the operator place a folded towel over the lock of the instrument, and in the standing position, bear heavily downward upon this pad with one hand, while traction was made in the ordinary way with the other. Osiander sought here, by applying two forces—one downward and the other downward and forward—to cause, as a resultant of these, an actual extractive force in the direction of the pelvic canal.

In setting forth what is known to me as to instruments intended to draw the head through the pelvis by a force applied in a direction perpendicular to the plane of the pelvis at which the progress of the head is opposed, perhaps I cannot follow a more natural course than to give an outline of my own efforts to construct a direct traction instrument. The first fact which struck my attention was that the pelvic curve of most forceps was not in correspondence with the actual curve of the pelvis. It seemed advisable, therefore, as a first step toward producing a direct traction instrument, that the second curve should be made to correspond with that of the pelvis. This being done by a proper shaping of the blades and shafts, it became evident that the line of traction fell far below the plane in which, in the ordinary methods of forming the instrument, the handles would lie. Accordingly these were depressed so as to form a decided angle with the shanks, so that the line of the handles should be parallel to a line drawn perpendicular to the axis of a cylinder of about the diameter of the head, held within the grasp of the forceps.

I may make clearer my meaning by means of this drawing, A B representing the blades and shanks curved as the pelvis. Now, in place of following the ordinary methods, and forming the handles in the direction of the dotted lines, C C, they were depressed to the point D, to such a degree that the plane of the handles, D C, should be parallel to a line perpendicular to the face of a cylinder, E E, held in the tractile grasp of the blade, A B.

I supposed that instruments so formed would possess three decided advantages: first, facility of introduction and easy coaptation to the head; second, the status of the handle relatively to the blades would cause the latter to give some idea of the line, perpendicular to the resisting circle of the pelvis, in which traction should be made; in other words, the direction in which the handles were pointing would indicate the direction in which traction on them should be effected; third, traction in the line of the handles would be perpendicular to the plane of resistance—that is, direct. And upon trial of instruments made after this plan upon a model, they held very well when the head was seized in a long or oblique diameter, and well also when applied over the sides of the head. In the latter position, however, the hold was only firm when traction was carefully made in the line of the handles. This could be done only by vigilantly guarding the ends of the handles near the lock from rising upward (toward the points of the blades) and by preventing a corresponding depression of the opposite ends.

It will be seen by this model that traction made at any points on the handles in a direction parallel to the axes of the blades tends to draw these points into line with the point or place of resistance on the blades, and experience at once showed that unless this tendency was successfully resisted, there was a great liability of the clams, when the head was grasped over the ears, to slip off, as Chapman expressed it, "sideways." That is, the forceps yielding in the direction of the line of traction, slip off in a line about midway between a line representing the axis of the blades, and another perpendicular to that axis. This tendency to slip was so decided as to condemn the instrument. In fact, in two cases in practice it slipped over the head when low down, in the manner detailed, after successful delivery through the superior strait, although in the hands of expert obstetricians. It became necessary, therefore, to alter the instrument so as to obviate the tendency to slipping. The first plan that occurred to me was to extend the lower rim of the blades farther backward, as in the forceps of Johnson, Davis and others, in order that the parietal protuberance should present an obstacle to the slipping off of the instrument in the direction stated. Careful test of forceps, provided with such a curve, demonstrated that the hold obtained by the widening of the blades was yet insecure. I then thought of pulling by a thong passed through the fenestræ imitating in this, as I believe, some of the older writers. This plan on the model gave quite satisfactory results, but practically it was not of much value, for in order to the successful use of the thong, it was necessary that it be pulled upon in a line farther back, than the perineum would admit of in the majority of cases. I had, a year ago, noticed the peculiar supplementary handle of the forceps of Dr. Hobbs, jutting downward from, and perpendicular to, the ordinary handles, and a trial of his instrument on the model, showed quite a firm hold. I began to conceive that this handle was of value for other reasons than that it presented an opportunity for powerful traction. It then occurred to me that having, as we do, a connection between the handles and the blade by means of two stout steel rods, the shanks, it ought to be practicable to draw on the head in the desired direction. I pictured to myself the above diagram.

A B being the blades of my forceps, A C the shanks, and C D the handle. Now, if a bar were firmly attached to the extremity of the handle at D, extending perpendicular to C D to the points E and F, we should be able to make the following demonstration: Let G represent the point of resistance, and in our experiment, a pivotal point. Now, if traction be made at E, the tendency of the apparatus would be to assume the position indicated in the dashed lines H K A. On the contrary, if traction were made at F the apparatus would tend to move into the position of the dotted lines M L K. Now, therefore, as a force at E moves the handle C D in one direction, and a force at F moves it in a contrary direction, there must be some point between the points E and F at which the handle, C D, will move neither in the one nor the other direction, as the point N. *This point would be the point of direct traction.*

e f g, horizontal and perpendicular portions of the traction-handle. The shanks, with the conjoined handles, c d g, forming a steel yoke spanning the projection of sacrum, coccyx and perineum, by means of which direct traction may be made in the line of a b, striking the traction handle at b, through the center of the operator's hand.

BARTLETT'S DIRECT TRACTION FORCEPS.

I had, therefore, only to attach to my forceps, a handle like that of Dr. Hobbs, and of such length as to enable the hand to grasp it at the point of direct traction determined by trial, to perfect my instrument—to make it what I had proposed to myself in the beginning, a direct traction forceps. I had this handle constructed so as to be instantly attached or removed, and in no wise to interfere with the manipulation of the handles. The practical question now was whether the new supplementary handle would successfully overcome the difficulty of slipping off sideways of the blades. Please to give your attention while I test the instrument on the model. You see, without the new handle, when traction is made in something of the old way, as in the ordinary forceps, the blades slip away from the head with a facility which is surprising; whereas, when the handle is applied, the tendency to rising upward and slipping of the blade is entirely overcome, and though having an unusually high curve, they hold with the tenacity of the straight forceps. In the use of these instruments, one acquires a tact which enables him to feel when the hold is insecure, or the reverse. When my forceps are applied without the new handle, the *feel* of the hold is suggestive of great insecurity; whereas, when the new handle is applied, the sensation imparted to the hand is that of an inflexible, unyielding grip.

Observe that the point at which it is necessary to apply the traction force in our illustration, varies with the portion of the surface of the blades at which the resistance actually exists. Thus, if the head be grasped by surfaces of the blade near the end, the point of direct traction rests farther below on the handle than when it is grasped nearer to the shanks of the instrument. Occasionally the head is seized by the lower rim of the forceps only, and in this case the point of traction falls lower down on the handle than if the points of resistance were on both rims. It will thus be seen that the point of direct traction varies on the traction handle an inch or more, but in practice it is thought that this will make no difference, provided that a point be chosen at which the traction is the lowest that might be necessary.

It is interesting in this connection to note that in practice the actual point of direct traction on the handle may be determined in any given case by trial. Thus; if a piece of tape, a foot in length, be looped on to the traction handle far toward its junction with the ordinary handles, and traction be made from its free extremity, it will be observed that the angles formed by the tape and the traction handle are not right angles, and that the obtuse angle faces that end of the handle farthest from the tape.

Whereas, if the thong be looped on far toward the other, free end of the handle, and traction be then made by it, the obtuse angle then formed faces in the contrary direction. And it will be found that the degree of deviation of the obtuse angle from a right angle, in either case, may be increased by carrying the tape yet farther toward that extremity of the handle to which it is then the nearest, and that on the contrary, the obtuse angle approaches more and more to a right angle according as the test cord is slipped along on the handle in the direction toward which the obtuse angle faces; so that presently we find it resting upon a point where the obtuseness of the angle has vanished and the tape and handle stand at right angles. *This is the exact point of direct traction for the resistance offered at the moment of trial.* It should be added that the determination of this point may be effected in less time than has been occupied in the recital of this account of the method of determining it.

In the use of this instrument it is sometimes desirable to bind the handles together, not only for the reasons which suggest this procedure in ordinary forceps, but that the whole instrument may be allowed to rotate with the head, if need be, in the loose grasp of the traction hand, free to turn on the admirable swivel formed by the joints of the forearm. Now, that compression may be completely under the control of the operator—instantly applied, regulated or removed—the following device has been adopted: The handles are bound loosely in the usual way and place, with a suitable thong of lace leather, including in the ligature the horizontal plate of the supplementary handle resting immediately beneath the ordinary handles. To apply and regulate compression, a suitable wedge of wood is adjusted under the thong *i. e.* between it and the traction handle plate, with the effect of tightening the ligature to any desired extent. To diminish compression, or to remove it entirely, the wedge is to be retracted, or withdrawn.

I should not omit to state that in the use of the forceps here described, more especially when the head is above the superior strait, it would be desirable, if not absolutely necessary, that the patient be in the English position, on the side. For the traction, the woman being on the back, would, in a high application, be so nearly downward that it could not be effectively applied. As to the great majority of practitioners in this country, the application of the forceps with the patient on the side would be novel and awkward, it would seem better to apply them in the dorsal position, and then place the patient in the English attitude.

THE HOBBS FORCEPS.

I desire next to call your attention to two other direct traction instruments, both of which were constructed before the one which I have just described. The more recent of these is that of Dr. John O. Hobbs; the earlier, that of Dr. Tarnier, of Paris. Dr. Hobbs modeled the blades of his instrument, in their general shape, after those of R. W. Johnson and Tarnier; they have a pronounced perineal curve. To make his forceps more effective, he added the very ingenious handle, to which reference has been made. This handle—a device which, as I believe, will mark an era in the construction of the forceps—not only enables the operator to pull with ease and force, but it, as demonstrated above, by lowering the point of traction, tends to make it direct.

THE TARNIER FORCEPS.

I now call your particular attention to the peculiarities of Tarnier's forceps. The shafts and blades have the old perineal curve, and reach quite high into the pelvis ; the handles are used as those of any other forceps, *except that they are not intended for extraction ;* they are provided with a screw, by which they may be held closed upon the head. The distinguishing peculiarity of the instrument is this supplementary piece, by which traction is made. It is a properly shaped bar of steel, provided at one end with diverging branches intended to hook into the shank end of the blades, while the other end, curved downward, has attached to it a bar by which traction may be made with both hands. Dr. Tarnier, in the addition of the tongue or hook-piece to his forceps, had evidently despaired of utilizing the shanks as a means of traction. He used the rod with the intention of drawing it as directly backward as the perineum would permit; and remark particularly that the cross-bar is not attached to the traction tongue close to the handles, but that it is bent downward an inch (2.5 ctm.) or more before this attachment is made. The depression downward of the bar is not, as some suppose, simply to remove it to a convenient distance from the handles, but to give to the instrument the power of a direct tractor.

The blades of Tarnier's forceps are not intended to be held together by the hand, but by means of the screw. One object of this modification is to allow the handles and the entire forceps liberty of motion, so that the natural movements of rotation, etc., of the head may take place without that restraint which the hold of the hand on the handles might occasion. Dr. Tarnier illustrates how these movements of the head are imparted to the forcep-handles on a manikin, the pelvic cavity of which is provided with resilient rubber walls so that the head in passing through it undergoes the ordinary movements incident to the mechanism of labor. We have not at hand a manikin of suitable kind, but the capability of movement may be perfectly shown by passing the blades grasping a model head through any suitable compressing surfaces, the irregular faces of which, like the planes of the pelvis, invite adaptive changes in the "position" of the body passing. Thus, if one, while seated, grasp the blades of the Tarnier forceps, closed upon a model head, between his apposed legs just below the knees, and draw upon the cross bar so as to cause the head to emerge between the thighs just above the knees, he will observe that the handles of the index forceps indicate the changes in rotation imposed on the head by the resilient walls offering it resistance. If the handles of my instrument in like manner be secured by a thong or elastic, and traction be made upon the traction handle by a loosely grasping hand, something of the same facility of movement is observed as in the original index forceps. I may here add, parenthetically, that I have taken the liberty to correct the translation of the words which Tarnier employed to designate his forceps, *Forceps à aiguille*, so that the idea embodied in the French may be retained in the English, the strictly literal rendering of the word *aiguille*, as usually translated, destroys its significance.

Though this forceps is complicated and awkward it is said to be readily applied and very efficient. It certainly holds admirably, and is a direct tractor. It would seem to me, however, in view of the manner in which the traction is made in my instrument, through the shanks, that there is no necessity for the hooked tractor of Tarnier.

It may and doubtless will be objected by some that there is no need of a powerful forceps—that there is power enough in any of the standard instruments to deliver, where delivery is possible. It is true that in the majority of cases, especially with the head low down, the ordinary style of forceps answers very well, but it is not to be denied that occasionally cases occur, and not very infrequently, in which the practitioner finds his strength exhausted, and feels the necessity of calling in assistance. At other times, the aid of the inexperienced, as of the nurse or husband must be invoked. Occasionally it happens that, through sheer exhaustion of the medical attendant, a failure to deliver occurs, and the perforator is brought into use. Any instrument which lessens the labor of forceps delivery, is of marked advantage to the obstetrican and the parturient, the more especially when the diminution of force required is effected by causing traction in the true line, and thus avoiding unnecessary pressure upon the tissues of the mother, and needless compression of the head of the child.

Recently these forceps were used in a case in which their efficiency was so strikingly shown, that I may briefly refer to it. Dr. F. Henrotin employed them in the delivery of a woman whom he had attended in four previous labors; in three of these, using the forceps, and on the other occasion turning, and in each instance experiencing great difficulty because of a narrowing of the conjugate, and a tilting forward of the womb, so that, as the woman sat, the abdominal parietes rested on the thighs. When called, at the recent confinement, Dr. Henrotin found that the woman had been in labor about twenty hours, and that the liquor amnii had been discharged fourteen hours. There was marked anteversion of the womb, and by introducing the hand, the head was felt, resting with its side above the pubic bones, and the vertex impinging against the promontory, the os being dilated to a diameter of two and one-half inches, and dilatable. Immediate recourse to the forceps was determined on. Dr. Henrotin introduced and adapted the blades readily, and delivered, with slight effort in less than fifteen minutes, to the surprise of himself and other physicians present.

### DISCUSSION.

Dr. Miller had been greatly interested in the able paper of the essayist. The forceps which the author had invented he thought very ingenious, but he feared a little too complicated and powerful for ordinary use. There was such a thing as a forceps being endowed with too much force; there was a limit to the force which can be invoked, beyond which it was not safe to go. A man of ordinary strength possesses all the force that prudence will allow. He believed that every power of the obstetric forceps should be controlled by an intelligent operator, and any change in the instrument which tended to lessen the required degree of intelligence was not a safe departure. The speaker once heard Lee say, at the close of an hour's lecture on the obstetric forceps, that, in his opinion, there had been no real improvement of the instrument since the days of Chamberlen. While he could not accept this extreme view, he believed it would be unfortunate for the woman if the forceps ever became such a perfect machine as to require less intelligence on the part of the operator in its use. Dr. Miller gave preference to a slight pelvic curve in the forceps. Above all, he thought it a matter of importance that the operator have a practical acquaintance with the particular instrument which he used. In conclusion, Dr. Miller said: The prudent operator will picture to his mind at all times the exact relations which exist between the head and pelvis and also the relations of his forceps to these parts. It was essential that his mind be occupied with these facts, and one objection which he raised to the instrument in point, was that the operator's attention was necessarily diverted from this by the manipulation of the complicated handles.

DR. ROLER said the paper had been an instructive one to him. Concerning the forceps which had been exhibited he feared it was endowed with too much power. In reality but little compression of the head was required in the great majority of cases. It was essential that the blades be applied exactly opposite each other, and when so applied the instrument could not slip. With regard to direct traction he thought that too much stress was laid upon it; we have an exaggerated idea of the pelvic curve. By directing the traction backward, with the hand bearing downward upon the instrument, as recommended by Osiander, and recently by Byford, one could exercise traction in the proper direction by means of the ordinary forceps.

DR. FITCH admired the ingenuity of the essayist as shown in his new forceps, but he thought them too complicated. The operator must at all times be conscious of the degree of pressure which he is exercising as he cannot be with the instrument in question. Notwithstanding the stress which had been laid upon the racking movement of the blades in a loose lock, he preferred the Siebold lock. Dr. Fitch called attention to the importance of one feature which is almost universally omitted in forceps, viz: the turning out of the tips of the blades, which enabled the operator to pass the instrument through a partially opened os with the greatest ease.

DR. JENKS thought the paper a very complete exposé of the mechanics of the different parts of the obstetric forceps. As a matter of history he desired to say that it was now generally admitted that Pugh added the pelvic curve to the forceps, fourteen years before it was mentioned by Smellie. The speaker dissented from Dr. Roler in that he had seen the forceps slip even when the blades were perfectly applied.

DR. NELSON said it was of the greatest importance that the compression of the head be intermittent for the safety of the fœtus, and for the integrity of the soft parts of both the fœtus and woman. He had known of one case of extensive sloughing of the scalp from too continuous traction, and two instances of destruction of the fœtus by pinching a loop of the umbilical cord, results that might have been avoided had the traction been intermittent.

DR. MERRIMAN said that one pair of forceps was not sufficient for all cases. On the whole he preferred Elliott's instrument, though he had known it to slip once or twice. As a rule a great curve of the instrument is not required, even in the high operation; the perineum can be pushed back so far as to permit traction to be made in the proper direction even with a nearly straight instrument. The speaker alluded to the very uncomfortable and awkward position which the operator was obliged to assume in making traction downward and away from his body in the high operation. Dr. Merriman also said there were cases in which forceps without handles were serviceable.

DR. SAWYER said that the uncomfortable position of the operator, spoken of by Dr. Merriman, could be obviated and many other advantages gained by placing the woman upon the side; he had adopted this position during the past two years and was now convinced of its superiority. It is a significant fact that the French and Americans who are confined to the back position feel the need of the perineal curve in the forceps, while the English, who place the woman upon the side, often use the straight forceps. As confirming the truth of what Prof. Miller had said, that an operator uses that instrument best with which he is the best acquainted, it was interesting to recall the very important results in Dr. Hamilton's practice, in Falkirk. This gentleman uses only the Zeigler straight forceps, and, what is strangest of all, he applies the instrument in the antero-posterior diameter of the superior strait.

DR. EARLE said there were undoubtedly cases where extrem force was required to complete delivery. He spoke of one case where the combined strength of both Dr. Fitch and himself was called in requisition.

In closing the discussion, Dr. BARTLETT said that the objection that the instrument was complicated was rather apparent than real. While in simple forceps delivery he would not select such an instrument, in a difficult case, he believed it would be more easy of application and more efficient in action than the ordinary forceps. Nothing but prolonged experience would give that *tactus eruditus* which should determine just what amount of downward pressure, and what measure of traction would produce a resultant force sufficient to counteract the errors of construction of any given instrument. Whereas the mode of using the forceps he presented, was readily understood, and it being properly applied, and the hand placed over the proper point of the traction handle, a novice would hardly pull in the wrong direction.

Imperfect description on his part had probably led to a misapprehension as to the mode and measure of compression to be used with the instrument. He called attention to the fact that the handles were so arranged that if the operator preferred, he might make compression with his hand as in the ordinary forceps. The thong and wedge attachment were devised with the express intention of placing the compression completely under the control of the operator ; by their use the compressing force could be applied, regulated or removed in an instant.

It had been said that it was essential in order to the firm hold of the forceps that the blades be applied the one exactly opposite to the other. So far as the instruments with a rigid lock were concerned, the statement was true, but it required only a glance at the model head held in the racking blades of the Ziegler forceps to show that the remark did not apply to instruments of that class.

In regard to the pelvic curve, there should be no discrepancy of opinion, as it might be said to be a fixed quantity, and no less, the speaker ventured to say, than that of the forceps he recommended.

It had been stated that higher curved instruments were not needed because the perineum may be so far pushed back toward the coccyx as to render possible the employment of ordinary instruments. He would call attention to the fact that in this procedure the forceps acted as a powerful lever, and that, while the blades might retain their grasp on the head, in such a manœuvre, and the perineum be forced backward, the contrary might happen, namely: the perineum might *not* yield, and the blades might be forced from off the head.

Dr. Bartlett called attention to the fact that in the use of the straight forceps antero-posteriorly, the blade of the instrument toward the sacrum while holding the head in its grasp, presented a curve very nearly corresponding to that of the parturient canal.

Referring to the trouble experienced in introducing the second blade, which Dr. Fitch had mentioned, Dr. Bartlett stated that in the ordinary dorsal position for forceps delivery, the thigh of the mother sometimes interfered with the free movement of the handle of the instrument laterally. In such cases he had the thigh raised to a right angle with the body, the assistant resting the leg over his shoulder.

## Figures and Tables

**Figure f1:**
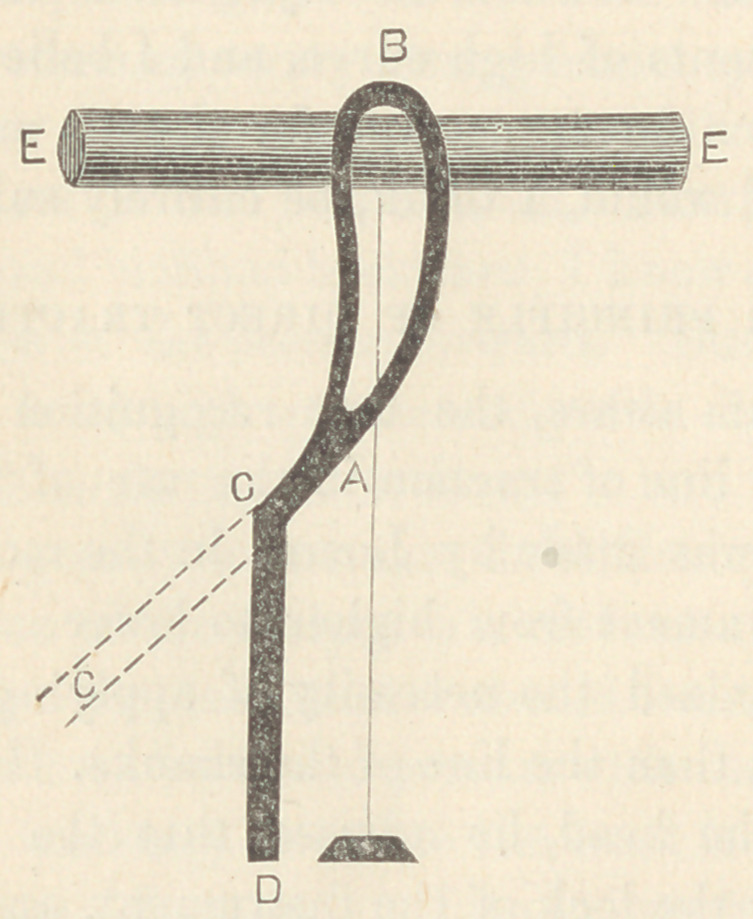


**Figure f2:**
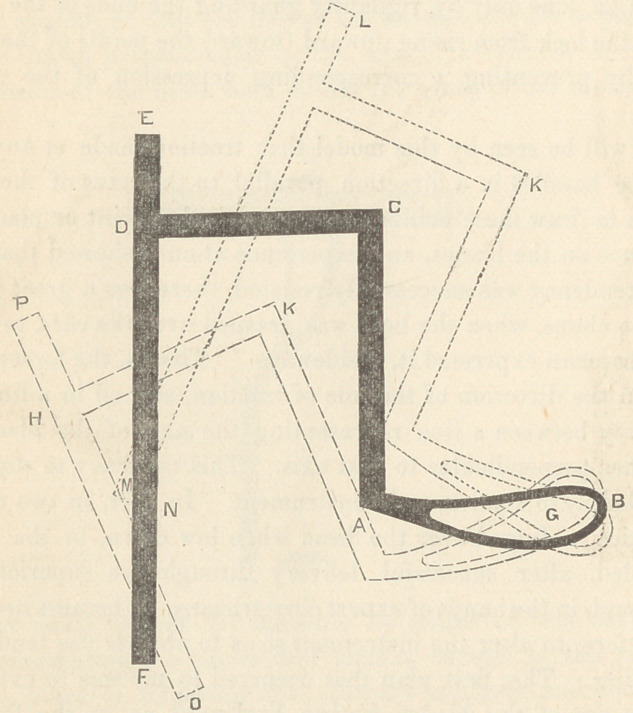


**Figure f3:**
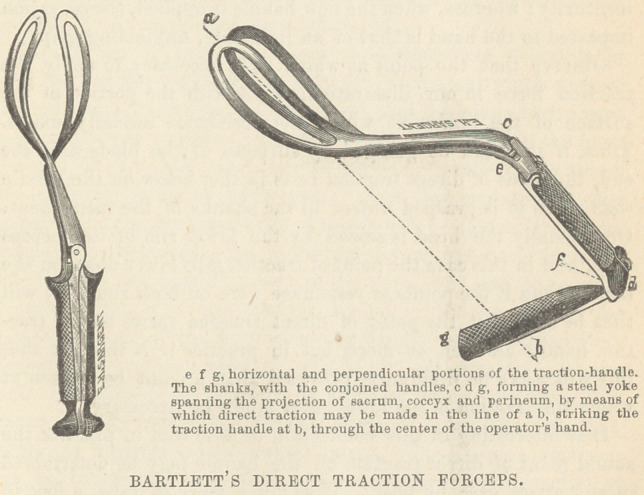


**Figure f4:**
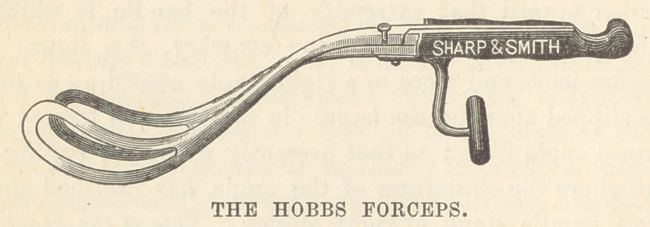


**Figure f5:**